# Oral Health in Young Adults: The Impact of Socioeconomic Factors and Health Literacy

**DOI:** 10.3390/ijerph22091407

**Published:** 2025-09-09

**Authors:** Lene Marita Steinvik, Gro Eirin Holde, Linda Maria Stein

**Affiliations:** 1 Department of Clinical Dentistry, Faculty of Health Sciences, UiT The Arctic University of Norway, 9037 Tromsø, Norway; gro.e.holde@uit.no (G.E.H.); linda.stein@uit.no (L.M.S.); 2 The Public Dental Health Service Competence Centre of Northern Norway, 9271 Tromsø, Norway; 3 Oral Health Centre of Expertise in Rogaland, 4016 Stavanger, Norway

**Keywords:** dentistry, health literacy, health promotion, health systems, Norway, oral health, quality of life, socioeconomic factors

## Abstract

Oral diseases remain prevalent, although most of them can be prevented. Oral health inequities represent a critical problem of social injustice worldwide. Health literacy has emerged as a potential determinant of oral health disparities, comparable in impact to socioeconomic factors. This study explored the impact of socioeconomic factors and health literacy on oral health in young adults in Norway using data from the third wave of the Fit Futures study (n = 705), which included questionnaires and clinical oral examinations. Measures included self-reported oral health, oral health-related quality of life (OIDP-8), caries experience (DMFT), gingivitis (BOP), health literacy (HLS-Q12) and socioeconomic factors. Multivariable logistic regression analyses showed that lower health literacy was associated with poorer self-reported oral health (OR: 0.95, 95% CI: 0.93–0.98) and lower oral health-related quality of life (OR: 0.94, 95% CI: 0.90–0.98). Higher health literacy was associated with a greater caries experience (OR: 1.05, 95% CI: 1.01–1.09). Lower educational attainment was associated with less favorable outcomes across all oral health measures (OR: 1.7–2.6, all *p* < 0.05). These findings suggest that both health literacy and education influence oral health. Interventions aimed at enhancing health literacy and reducing barriers should be tested to empower young people and support their long-term oral health.

## 1. Introduction

Oral health is considered an integral part of health and well-being, contributing to essential oral functions such as eating, speaking, smiling and social interactions [1]. This also includes physical and psychosocial aspects that affect a person’s quality of life and ability to participate in society [2]. The Nordic countries provide a gold standard for welfare societies with policies to ensure high social security and equality for all citizens [3]. The welfare state provides access to free education and a social security system that supports individuals in need, including unemployment benefits, pensions, disability support and necessary healthcare. However, oral health services are not universal after the age of 18 [4].

In Norway, children and adolescents receive free, regular dental care until the age of 18 [4]. From the age of 19, young adults have historically transitioned into the adult patient group, which is responsible for paying out-of-pocket for dental services. Over the past few years, the Norwegian government has decided to extend young adults’ rights to subsidized dental services up to age 28 where they receive 75% discounted dental services [5,6]. Although this policy change was implemented after the data collection for this study, it reflects the growing recognition of the importance of oral health in young adulthood and the need to better understand the barriers and needs during this life-stage.

Despite strong public health initiatives and emphasis on oral health prevention within the dental services, national data show that oral diseases remain prevalent and continue to pose a public health challenge in Norway [4,7,8,9]. These preventive efforts have likely contributed to declining rates of dental caries and tooth loss over the past decades [7,8]. However, recent research points to increasing rates of initial caries in young adults and a notable prevalence of periodontal disease, which is uncommon for this age group [8,9,10]. These findings point to early signs of potential oral health problems for some young adults, which may be related to social inequality [11,12].

A recent review of inequalities in health and well-being in Norway found a high and increasing standard of living for most of the population, but with some significant and growing social and economic disparities [13]. Over time, the prevalence of oral diseases in a population also reflects society and the effectiveness of oral health policies for the population. Oral health outcomes often mirror broader societal inequities, and the persistence of preventable oral diseases among young adults may reflect underlying social gradients. These disparities likely stem from complex interactions between upstream determinants such as education and income, as well as more proximal factors, including behaviors, access, and individual capabilities.

In recent years, health literacy has been identified as an essential factor influencing health outcomes [14]. Health literacy is defined as “people’s knowledge, motivation and competencies to access, understand, appraise, and apply health information in order to make judgments and take decisions in everyday life concerning healthcare, disease prevention and health promotion to maintain or improve quality of life during the life course” [15]. For oral health, lower health literacy has been linked to less frequent use of dental services, poorer oral hygiene practices, poorer oral health outcomes and lower oral health-related quality of life [16,17,18,19,20]. The World Health Organization (WHO) has recognized health literacy as a determinant of health equal to the impact of traditional socioeconomic factors [21,22].

From a life-course perspective, oral health in young adulthood might influence long-term health and well-being trajectories [2,12]. Poor oral health during this age may cause pain, infection, and psychosocial challenges, potentially affecting educational attainment, careers and work participation [11,12]. Furthermore, young adulthood represents a transitional phase in life, often marked by increasing autonomy in health-related decision-making [23], which requires a solid foundation of health literacy. Despite the growing recognition of the role of both health literacy and socioeconomic factors in oral health [11,17], few studies have examined their combined impact on both subjective and clinical oral health outcomes. In addition, research on the young adult population in high-income countries is limited, particularly regarding how these relationships unfold within a universal welfare system like Norway’s.

Therefore, this study aimed to explore the impact of socioeconomic factors and health literacy on oral health in young adults in Norway.

## 2. Materials and Methods

### 2.1. Study Design

The Fit Futures study is a population-based health survey following adolescents from Northern Norway over the course of ten years [24,25]. All students attending their first year of upper-secondary school in Tromsø and the neighboring municipalities during 2010–2011 were invited to participate in the first wave of the Fit Futures study, FF1 (n = 1117), of which 1038 participated (92.9%, mean age 16.4, SD ± 1.2, 49.0% female). Recruitment was conducted through their schools, and data collection in FF1 took place during school hours. Transportation was arranged to facilitate participation. No formal sample size calculation was performed, as the Fit Futures study is a population-based cohort study. The aim was to include as many participants as possible from the target population. The second wave, Fit Futures 2 (FF2), was conducted two years later with no clinical oral measurements, except for clinical photos, which included clinical measurements of oral health. The third wave, Fit Futures 3 (FF3) was conducted in 2021–2022, ten years after the first wave. All previous participants in FF1/FF2 were contacted via phone or email. Those who had moved away from the region were offered reimbursement for travel expenses to facilitate participation. A total of 705 of the participants from FF1/FF2 participated in FF3 (61.1%, mean age 26.9, SD ± 1.2). This cross-sectional study is based on collected data from FF3.

The data collection in the Fit Futures study included extensive digital self-administered questionnaires and clinical health examinations, including clinical oral examinations and collection of biological samples. The present study utilized data from the self-administered questionnaires and clinical and radiographic examinations from the third wave, FF3. For the clinical and radiographic examinations of FF3, examiner calibration and inter- and intra-observer reliability assessments were performed [24].

All participants provided written informed consent before participating in the Fit Futures studies. The regional committee for medical and health research ethics approved the Fit Futures study (reference number 2009/1282) and the present oral health study (reference number 563627). The Norwegian Centre for Research Data reviewed and approved the current study and its data management (reference number 660941).

### 2.2. Measurements and Categorization

A summary of the measurements and categorization is presented in Table 1.

#### 2.2.1. Oral Health Outcomes

Oral health outcomes were assessed using both subjective and clinical oral health measures.

Self-reported oral health was assessed by asking “How do you consider your oral health?”, with response options on a 5-point Likert scale from “Very Poor (1)” to “Very good (5)”. For the analysis, the responses were categorized and further dichotomized (Table 1).

Oral health-related quality of life (OHRQoL) was assessed using the Oral Impacts on Daily Performance scale (OIDP-8) [26,27]. Participants reported the frequency of oral impacts over the past six months on a 5-point scale: “Never (1)”, “Less than once a month (2)”, “Once or twice a month (3)”, “1–2 times a week (4)”, and “Every day/almost every day (5)”. Responses were dichotomized according to whether the participant experienced any oral impact (options 2–5) or not (option 1) and summarized (range 0–8). The OIDP-8 sum score was further categorized and dichotomized for the analysis (Table 1). The internal consistency was acceptable (Cronbach’s alpha of 0.79).

Caries experience, defined as DMFT by the WHO [28], was measured using the DMFT index identifying the participants’ number of decayed (D), missing (M) or filled (F) teeth (T). Assessments included four bitewing radiographs, intraoral photographs, and clinical examination. DMFT score (range 0–28) was grouped into quartiles based on the DMFT distribution (Q1–Q4). For the analysis, Q4 represented “high caries experience” and Q1–3 “lower caries experience”.

Gingivitis was assessed via bleeding on probing (BOP) at four sites per tooth (buccal, distolingual, lingual, and mesiolingual), excluding the third molars, and recorded within approximately 20 s of probing. Participants with a BOP score ≥10% indicated gingivitis. Gingivitis was classified as localized (10–30%) or generalized (>30%) [29]. “Generalized gingivitis” (>30%) was compared to “no or localized gingivitis” (≤30%) in the analysis.

#### 2.2.2. Covariates

Age was recorded in years, and gender was recorded as “male” or “female”.

The use of dental services was assessed by asking the question, “Do you have regular visits to dental services?”, with six response options (Table 1). Responses were dichotomized into “Yes” (any frequency) and “No” (never or only for acute problems).

Health literacy was measured using the HLS-Q12 (17), a validated 12-item questionnaire assessing difficulty in accessing, understanding, appraising, and applying health information within healthcare, disease prevention and health promotion settings. The instrument derived from the European Health Literacy Survey Questionnaire [30,31]. Response options were “don’t know (0)”, “very difficult (1)”, “difficult (2)”, “easy (3)” and “very easy (4)”. “Don’t know” responses were treated as missing. Participants with >3 missing items were excluded. HLS-Q12 ranged from 12 to 48, with scores ≤33 indicating lower health literacy (Level 1) and scores >33 indicating higher health literacy (Level 2/3) [32,33]. The internal consistency was acceptable (Cronbach’s alpha = 0.77).

Educational level was based on the highest completed educational level. The variable was categorized and further dichotomized into “Lower education” and “Higher education” for the analysis (Table 1).

Financial situation was assessed by asking the question, “How do you evaluate your finances today?”, on a 5-point scale from “Very Poor” (1) to “Excellent” (5). Responses were categorized and dichotomized for the analysis (Table 1).

Work participation was assessed by asking the question: “Do you work/study?”, with ten response options (Table 1). Participants could choose more than one alternative. Responses were grouped into: “Working full-time” (1), “Partially working” (2–5), and “Not working” (6–10).

### 2.3. Statistical Analysis

Statistical analyses were performed using IBM SPSS Statistics version 29. Statistical significance was set at the 5% level. Descriptive statistics are presented as frequencies (n), percentages (%), and means (M) with standard deviation (SD).

Preliminary analysis included visual inspection of plots and histograms, as well as parametric and non-parametric correlations and cross-tabulations. Variable categorization was based on theoretical background and group distributions. The age range was 26–36 years (M = 26.9, SD ± 1.2), with 95.5% of participants within one standard deviation of the mean. Due to this narrow range and homogeneity, age was not expected to influence health literacy or oral health outcomes and was therefore excluded from the analysis.

Missing data occurred at a low frequency (0–3.7%), except for DMFT (7.2%), BOP (8.4%), OIDP-8 (5.1%) and HLS-Q12 (16.6%). For HLS-Q12, 91 participants (12.9%) had more than three missing items. The pattern of missing data appeared random. All missing cases were excluded pairwise in the analyses.

Group differences in health literacy scores (HLS-Q12) were assessed using independent samples *t*-tests and one-way ANOVA with post hoc Tukey HSD tests. While the HLS-Q12 showed statistically significant deviations from normality (Shapiro–Wilk *p* < 0.001; Kolmogorov–Smirnov *p* = 0.005), these were expected due to the large sample size. Further inspection confirmed approximate normality (Skewness = 0.02, Kurtosis = −0.52; mean = 5% trimmed mean = 35.3). To ensure robustness, sensitivity analyses were also performed using non-parametric tests (Mann–Whitney U and Kruskal–Wallis H), yielding comparable results (not tabulated).

Multivariable binary logistic regression was used to examine the associations between health literacy and four dichotomized oral health outcomes, while adjusting for socioeconomic factors and dental service use. Regression assumptions were met (tolerance > 1.0, VIF < 1.1, intercorrelations < 0.70). The assumption of linearity in the logit for the continuous predictor (HLS-Q12) was tested using the Box-Tidwell procedure and found to be satisfied (*p* > 0.05). Oral health outcomes were dichotomized due to their non-normal and often skewed distributions (DMFT, BOP, OIDP-8) and based on a conceptual focus on identifying participants with clinically meaningful impairments. This approach enhanced interpretability by contrasting those with poor vs. good oral health status. Ordinal predictors (educational level and financial situation) were treated as categorical variables using dummy coding.

## 3. Results

### 3.1. Descriptives

Sample characteristics and mean comparison of HL scores of control variables are presented in Table 2. Slightly more female (55%) than male participation in the third wave of the Fit Futures study. Most of the participants had higher education (53%), were working fulltime (57%), considered their financial situation as “good” (48%) and had regular use of dental services (72%). The mean health literacy score (HL score) was 35.3 (SD ± 6.0) (Table 2). When comparing mean HL scores, a noticeable difference was observed across educational levels, financial situation, and use of dental services. Specifically, primary education, characterized by a poor financial situation and irregular use of dental services, resulted in lower HL scores (all *p* < 0.001). HL scores did not differ significantly according to work participation.

Descriptive statistics for oral health outcomes and the comparison of mean HL scores are presented in Table 3. Most of the participants considered their oral health as “Good” (44%), closely followed by “Moderate” (41%). More than half (52%) reported no oral impacts on their daily performances, indicating minimal interference on their oral health-related quality of life. Half of the participants had a DMFT-score within the first and second quartile (Q1 and Q2) of the DMFT distribution, indicating less experience with caries disease. Additionally, 5% of the participants had no caries experience. Furthermore, almost all (85%) of participants had gingivitis, whereas approximately one third (31%) had generalized gingivitis. Although a large proportion of the participants reported favorable oral health outcomes, a subset of young adults in this sample considered their oral health “Poor” (12%), had three or more oral impacts (11%), and had high caries experience (18%).

When comparing mean HL scores, a noticeable difference was observed in HL scores for self-reported oral health, oral impacts on daily performance and caries experience (Table 3). For self-reported oral health, HL scores were higher for those reporting good oral health (36.2) than for those reporting average (34.5) or poor oral health (34.1), *p* = 0.002. Similarly, for oral impacts on daily performance, participants reporting ≥3 oral impacts had lower HL scores (33.3) than those reporting 1–2 (35.5) or no impacts (35.6), *p* = 0.020. For caries experience, measured by DMFT quartiles, the highest quartile (Q4) had higher HL scores (36.7) than those in the third quartile (Q3) (34.4), *p* = 0.020, while no significant differences were observed among the remaining quartiles.

### 3.2. Multivariable Logistic Regression

#### 3.2.1. Subjective Oral Health Measures

Participants with higher HL scores had reduced odds of considering their oral health to be “poor or moderate” and reporting 3 or more oral health impacts on daily performances. The findings revealed that for each increase in HL score, the odds of perceiving their oral health as “poor or moderate” decreased by 5%, and the odds of having three or more oral impacts decreased by 6% (Table 4). The effect on both outcomes was slightly reduced when adjusting for covariates. Participants with lower educational levels had 1.9 times higher odds of considering their oral health as “Poor or moderate” and 2.6 times higher odds of reporting three or more oral impacts than those with higher educational levels. Additionally, participants with irregular use of dental services had 3 times higher odds of reporting “poor or moderate” oral health than those who reported regular use of dental services.

#### 3.2.2. Clinical Oral Health Measures

Higher HL scores were associated with higher caries experience in this sample (DMFT 11–25). The findings revealed that for each increase in HL score, the odds of having the highest caries experience increased by 5% and the odds were slightly increased when adjusting for covariates (Table 5). Participants with irregular use of dental services had 3 times higher odds of having greater caries experience (DMFT 11–25) than participants with regular use of dental services. For gingivitis, there was a tendency towards having increased odds of generalized gingivitis per increase in HL score. However, the association between HL score and gingivitis among the young adults in this sample was not statistically significant. Nevertheless, educational level seemed to be influential for both clinical oral health measures. Participants with lower educational levels had 2.5 times higher odds of greater caries experience (DMFT 11–25), and 1.7 times higher odds of generalized gingivitis (BOP > 30), than participants with higher educational levels.

## 4. Discussion

### 4.1. Main Findings

Health literacy and educational level had a significant impact on subjectively and clinically measured oral health outcomes in this sample of young adults. While most participants reported “good” self-reported oral health measures and showed low levels of caries experience overall, a distinct subgroup among them reported poorer self-reported oral health, experienced multiple oral health impacts on daily performance, and displayed high caries experience and generalized gingival inflammation. These findings align with previous research [8,9] and support the growing evidence that young adults might not be as healthy as previously assumed [34].

Notably, gingivitis was highly prevalent in this sample, affecting nearly all participants. This highlights the importance of including both subjective and clinical data in oral health research. Moreover, the disconnect between this high prevalence of gingivitis and generally positive subjective oral health measurements may reflect limited awareness or understanding of early-stage oral disease among young adults. This further demonstrates the relevance of health literacy in health risk perception and care-seeking behavior.

### 4.2. Interpretation and Theoretical Framework

Consistent with previous studies, health literacy was associated with self-reported oral health and oral health-related quality of life [18,19]. Participants with higher health literacy scores had lower odds of reporting their oral health as “poor or moderate” and of experiencing multiple oral health impacts. This suggests that health literacy may influence not only oral health knowledge and behaviors but also how individuals perceive, interpret, and cope with oral health challenges. From a theoretical perspective, positive self-reported oral health and high OHRQoL may also serve as personal resources that buffer stress and promote health-supportive behavior, aligning with salutogenic models and the concept of sense of coherence [35,36,37].

Unexpectedly, higher health literacy scores predicted greater caries experience (DMFT 11–25), a finding that contrasts with previous research [38,39]. One possible explanation could be that individuals with more caries may have had greater exposure to dental services and received more chair-side oral health education. This could have led to higher reported health literacy, particularly at the interactive or critical levels described in Nutbeam’s model of health literacy [40]. Interactive health literacy involves advanced cognitive and social skills that enable individuals to engage with and apply health information to their circumstances, while critical health literacy, the most advanced level, refers to the ability to critically analyze health information in different contexts and use it to take greater control over life events and situations [40]. Through this line of thinking, health literacy is not only a determinant of oral health but also a consequence of engaging in the healthcare system. Nevertheless, oral health education should not be an advantage only for those who experience oral diseases. Ideally, health literacy should function as a tool for health promotion and disease prevention for all individuals [41].

Lower educational attainment was also significantly associated with poorer oral health outcomes across all oral health measures, including generalized gingivitis, higher caries experience, and poorer self-reported oral health and oral health-related quality of life. This was observed even though children and adolescents in Norway have equal access to free dental services until age 18 [4], and that higher education is tuition-free and widely accessible [3]. This demonstrates that equal access to dental services and education alone is not enough to reduce inequalities in oral health outcomes. Furthermore, this reinforces the role of education and its functions as a structural determinant shaping long-term health behaviors, attitudes, and resources, as described in the social determinant of health framework [11,42]. The persistent inequalities in oral health observed here may also reflect social and cultural factors, such as parental education and influence, intergenerational disadvantage, and systemic challenges faced by individuals with immigrant or minority backgrounds.

### 4.3. Public Health Implications and Recommendations

The current findings contribute to the growing evidence that health literacy is a modifiable and independent determinant of oral health outcomes [2]. In this study, health literacy was associated with three out of the four oral health measures, and these associations remained stable even after adjusting for socioeconomic factors. Nevertheless, health literacy does not exist in a vacuum. It is shaped by the broader social, structural and organizational contexts, including how health services communicate, engage with patients, and provide access to relevant information [15,21]. In Norway, the transition to adulthood often requires young people to navigate dental services more independently, which can expose disparities in health-related decision-making, especially among those with limited socioeconomic resources.

These findings also suggest the need to integrate health literacy into oral health promotion strategies that reflect both individual and systemic perspectives. Oral health promotion in accessible, inclusive, and culturally sensitive ways can help support young adults in managing their oral health [43,44,45]. Interventions, such as school-based oral health programs, social media campaigns, and digital tools tailored for young people, can help support young adults in managing their oral health. For example, social media campaigns on popular platforms among young people, such as Instagram or TikTok, can be particularly effective for oral health engagement, as they offer interactive content and peer discussions [43]. Dental professionals can play a key role in these efforts [44]. By focusing on empowerment and providing clear, understandable health information, they can help young people make better oral health choices.

Health literacy efforts should be integrated into broader public health strategies that address structural barriers, such as affordability, access to care, and social inequality. Interventions that enhance health literacy, while addressing structural barriers, service availability, and educational attainment, may be more effective in improving oral health outcomes among young adults. Although the study population did not directly benefit from it, the recent policy change in Norway, which extends subsidized dental services up to age 28, represents a step toward reducing barriers and enhancing oral health equity.

### 4.4. Strengths and Limitations

This study draws on data from the Fit Futures cohort, which is an extensive population-based study with a high participation rate. The use of both self-reported and clinically assessed oral health measures enabled a more comprehensive understanding of oral health outcomes in young adults, as well as the associations with health literacy and socioeconomic factors in a welfare state such as Norway. By focusing on young adults, this study addresses an otherwise understudied life-stage in terms of oral health research.

While the study was conducted in Northern Norway, the city of Tromsø and the more rural neighboring municipalities reflect the demographic and socioeconomic diversity seen in both urban and rural populations across the country. Although Northern Norway has unique geographic features, the population in this region includes a mix of cultural and socioeconomic backgrounds similar to those in other parts of the country, making the findings broadly relevant. However, as the ethnicity was not recorded, we can not provide any interpretations on the generalizability of this sample when it comes to ethnicity. Additionally, the region of Northern Norway has historically faced challenges in recruiting oral health professionals, which may have impacted the availability of oral health services and, consequently, oral health outcomes.

Several other limitations should be acknowledged. The cross-sectional design restricts the ability to determine causal relationships between health literacy, socioeconomic factors, and oral health outcomes. Health literacy was only measured at the third wave (FF3), which limits the ability to analyze changes over time or draw causal conclusions. However, at the time of the first wave (FF1), health literacy had not yet been widely recognized as a public health priority in Norway. Additionally, homogeneity of the age cohort could be considered as a limitation in this study, particularly as health literacy is a dynamic ability often associated with increasing age and experience over time [15].

While the HLS-Q12 is a validated general health literacy instrument, it is not oral health-specific. The Norwegian version of the HLS-Q12 has demonstrated acceptable validity and reliability in prior studies [30], but its psychometric properties have not yet been extensively validated among Norwegian young adults. Similarly, the Norwegian version of the ODIP-8 has been adapted, validated and tested for use in the Norwegian context, and its translation has previously been used in national surveys [27,36]. However, a formal validation of ODIP-8 among Norwegian young adults remains limited. These limitations may have affected the measurement accuracy and sensitivity of the HLS-Q12 and the ODIP-8.

Furthermore, the categorization and dichotomization of oral health outcomes and other variables may have resulted in some loss of information and introduced misclassification bias. Additionally, self-reported measures are subject to recall and social desirability bias, particularly for subjective outcomes such as self-reported oral health, use of dental services and OHRQoL. Measurement bias may also have occurred during the clinical examinations, especially in the assessment of BOP, as probing pressure was not standardized across examiners. Although substantial calibration efforts were made during data collection, inter-examiner variability cannot be ruled out.

In terms of selection bias, more males were lost to follow-up between earlier waves and FF3. In addition, the initial sampling of the Fit Futures cohort, only students enrolled in upper secondary education were included in the first wave. This may have led to an underrepresentation of individuals with lower educational attainment. Furthermore, individuals with the lowest health literacy are typically less likely to participate in or complete self-administered questionnaires [46], which may have led to an overestimation of health literacy in the final sample. These considerations in terms of representativeness and sampling need to be taken into account when reviewing the findings.

### 4.5. Future Research

Future studies should employ longitudinal designs to better understand the causal pathways between health literacy and oral health outcomes. It would also be valuable to differentiate between active disease and past disease when using indices like DMFT. The DMFT score indicates the number of decayed, missing, and filled teeth, but the measurement does not distinguish between active caries (DT, decayed teeth) and past disease or treatment (FT, filled teeth). This distinction could provide clearer insights into the current disease burden and its association with health literacy. The finding that health literacy is linked with both subjective and clinical measures further demonstrates its potential use in clinical screening and health communication strategies. Moreover, future research should consider using oral-health-specific health literacy instruments to increase measurement precision. Greater emphasis should also be placed on understanding how health literacy interacts with digital health communication, service accessibility, and the effect of interventions on oral health and oral health-related behaviors.

## 5. Conclusions

While the overall oral health status was favorable among most young adults, the distribution of oral disease was uneven, with a smaller proportion bearing a greater burden of disease. Health literacy and educational level had an impact on oral health among young adults in this sample. Interventions aimed at enhancing health literacy and reducing barriers should be tested in future research studies to empower young people and support their long-term oral health.

## Figures and Tables

**Table 1:** Summary table of measurements and categorization.

Variable	Measurement	Response Options	Categorization	Dichotomization
Oral health outcomes				
Self-reported oral health	“How do you consider your oral health?”	Very Poor (1)	Poor (1–2)	Poor or moderate (1–3)
		Poor (2)		
		Moderate (3)	Moderate (3)	
		Good (4)	Good (4–5)	Good (4–5)
		Very good (5)		
OHRQoL	Oral impacts on daily performance scale (OIDP-8)	0–8 oral impacts	0 oral impacts	0–2 oral impacts
			1–2 oral impacts	
			≥3 oral impacts	≥3 oral impacts
Caries experience	Decayed, missing or filled teeth (DMFT)	0–28 DMFT	Q1 (0–3 DMFT)	Q1–3 (0–10 DMFT)
			Q2 (4–6 DMFT)	
			Q3 (7–10 DMFT)	
			Q4 (11–25 DMFT)	Q4 (11–25 DMFT)
Gingivitis	Bleeding on probing (BOP)	0–100% bleeding on probing	No gingivitis (<10%)	No or localized gingivitis (≤30%)
			Localized gingivitis (10–30%)	
			Generalized gingivitis (>30%)	Generalized gingivitis (>30%)
Covariates				
Age		26–36 years		
Gender		Male		
		Female		
Health literacy	European Health Literacy Survey Questionnaire (HLS-Q12)	12–48 score		
Use of dental services	“Do you have regular visits to dental services?”	No, never		No
		No, just for acute problems		
		Yes, more than once a year		Yes
		Yes, every year		
		Yes, every second year		
		Yes, more than two years apart		
Educational level	“What is the highest level of education you have completed?”	Primary education (1)	Primary education (1)	Lower education (1–3)
		Occupational high school (2)	Secondary education (2–3)	
		High school (3)		
		University < 4 years (4)	Higher education (4–5)	Higher education (4–5)
		University ≥ 4 years (5)		
Financial situation	“How do you evaluate your finances today?”	Very Poor (1)	Poor (1–2)	Poor (1–2)
		Fair (2)		
		Average (3)	Average (3)	Good or average (3–5)
		Good (4)	Good (4–5)	
		Excellent (5)		
Work participation	“Do you work/study?”	Full-time (1)	Working full-time (1)	
		Part-time (2)	Partially working (2–5)	
		Student (3)		
		Parental leave (4)		
		Sick leave (5)		
		Unemployed (6)	Not working (6–10)	
		Work assessment allowance (7)		
		Disabled (8)		
		Social welfare (9)		
		Other (10)		

**Table 2:** Sample characteristics and mean comparisons of HL scores.

Sample Characteristics	n (%)	HLS-Q12		
		n	Mean (SD)	*p*-Value
Total	705 (100)			
Age, mean (SD)	26.9 (1.2)	588	35.3 (6.0)	0.227 *
Gender				0.056
Female	388 (55.0)	330	35.7 (6.1)	
Male	317 (45.0)	249	34.7 (5.7)	
Educational level				<0.001
Primary education	41 (5.8)	30	32.1 (6.0)	
Secondary education	266 (37.7)	220	34.7 (5.8)	
Higher education	372 (52.8)	338	36.0 (6.0)	
Missing	26 (3.7)			
Financial situation				<0.001
Poor	89 (12.6)	76	33.7 (6.1)	
Average	251 (35.6)	219	34.5 (6.0)	
Good	339 (48.1)	293	36.3 (5.8)	
Missing	26 (3.7)			
Work participation				0.120
Not working	46 (6.5)	36	33.4 (5.3)	
Partially working	233 (33.1)	205	35.2 (6.2)	
Working fulltime	399 (56.6)	347	35.5 (5.9)	
Missing	27 (3.8)			
Use of dental services				<0.001
No, never or only acute	172 (24.4)	143	33.8 (5.3)	
Yes	507 (71.9)	445	35.8 (6.1)	
Missing	26 (3.7)			

* Pearson’s correlation was performed for this variable.

**Table 3:** Oral health outcomes and mean comparisons of HL scores.

Oral Health Outcomes	N (%)	HLS-Q12		
		n	Mean (SD)	*p*-Value
Self-reported oral health				
Good	310 (44.0)	274	36.2 (5.9)	0.002
Moderate	287 (40.7)	244	34.5 (6.1)	
Poor	81 (11.5)	69	34.1 (5.7)	
Missing	27 (3.8)			
Oral health-related quality of life				
ODIP-8, mean (SD)	1.0 (1.6)			0.019
0 oral impacts	368 (52.2)	327	35.6 (6.0)	
1–2 oral impacts	223 (31.6)	189	35.5 (5.9)	
≥3 oral impacts	78 (11.1)	65	33.3 (6.1)	
Missing	36 (5.1)			
Caries experience				
DMFT, mean (SD)	6.6 (4.6)			0.015
Q1, 0–3 DMFT	178 (25.3)	151	34.9 (6.3)	
Q2, 4–6 DMFT	175 (24.8)	143	35.7 (5.8)	
Q3, 7–10 DMFT	171 (24.3)	141	34.4 (5.8)	
Q4, 11–25 DMFT	130 (18.4)	104	36.7 (5.9)	
Missing	51 (7.2)			
Gingival status				
BOP, mean (SD)	26.2 (12.8)			0.630
BOP < 10%	48 (6.8)	38	35.8 (6.5)	
BOP 10–30%	381 (54.0)	317	35.1 (6.1)	
BOP > 30%	217 (30.8)	178	35.6 (5.6)	
Missing	59 (8.4)			

**Table 4:** Logistic regression analysis for the association between health literacy score and subjective oral health measures.

	Subjective Oral Health Measures			
	Self-Reported Oral Health Poor or Moderate vs. Good		Oral Impact on Daily Performance ≥3 Impacts vs. 0–2 Impacts	
	OR (95% CI)	*p*-Value	OR (95% CI)	*p*-Value
HL score, unadjusted	0.95 (0.93–0.98)	<0.001	0.94 (0.90–0.98)	0.006
HL score, adjusted *	0.97 (0.94–0.99)	0.027	0.95 (0.90–0.99)	0.018
Gender—male vs. female	1.32 (0.93–1.89)	0.122	1.14 (0.67–1.96)	0.625
Education—lower vs. higher	1.89 (1.32–2.71)	<0.001	2.59 (1.48–4.53)	<0.001
Financial situation—poor vs. good or average	1.05 (0.61–1.80)	0.860	0.98 (0.46–2.09)	0.962
Use of dental services—irregular vs. regular	2.95 (1.90–4.58)	<0.001	0.88 (0.47–1.63)	0.681

* Adjusted for gender, education, financial situation, and use of dental services.

**Table 5:** Logistic regression analysis for the association between health literacy score and clinical oral health measures.

	Clinical Oral Health Measures			
	DMFT Q4 vs. Q1–3		BOP >30 BOP vs. ≤30 BOP	
	OR (95% CI)	*p*-Value	OR (95% CI)	*p*-Value
HL score, unadjusted	1.05 (1.01–1.09)	0.010	1.01 (0.98–1.04)	0.455
HL score, adjusted *	1.06 (1.02–1.10)	0.005	1.03 (0.99–1.06)	0.134
Gender—male vs. female	0.88 (0.56–1.39)	0.574	1.14 (0.78–1.67)	0.498
Education—lower vs. higher	2.48 (1.57–3.94)	<0.001	1.68 (1.14–2.47)	0.009
Financial situation—poor vs. good or average	0.77 (0.39–1.55)	0.469	0.70 (0.40–1.25)	0.226
Use of dental services—irregular vs. regular	0.85 (0.49–1.48)	0.568	1.92 (1.24–2.95)	0.003

* Adjusted for gender, education, financial situation, and use of dental services.

## Data Availability

Data sharing is not applicable to this article. The original contributions presented in this study are included in the article. Further inquiries can be directed to the corresponding author.
